# Biomechanical Analysis of Non-Metallic Biomaterial in the Manufacture of a New Knee Prosthesis

**DOI:** 10.3390/ma14205951

**Published:** 2021-10-10

**Authors:** Miguel Suffo, Carlos Revenga

**Affiliations:** 1 Department of Mechanical Engineering and Industrial Design, High Engineering School, Universidad de Cádiz, Campus Río San Pedro s/n, 11510 Puerto Real, Spain; 2 Orthopedic and Trauma Department, San Juan Grande Hospital, 11003 Jerez de la Frontera, Spain; c_revenga@hotmail.com

**Keywords:** non-metallic knee prosthesis, ULTEM^TM^ 1010 biomaterial, additive manufacturing, biomechanical design, two-component knee prosthesis, live/dead

## Abstract

The increase in the number of revision surgeries after a total knee replacement surgery reaches 19%. One of the reasons for the majority of revisions relates to the debris of the ultra-high molecular weight polyethylene that serves to facilitate the sliding between the femoral and tibial components. This paper addresses the biomechanical properties of ULTEM^TM^ 1010 in a totally new knee replacement design, based on one of the commercial models of the Stryker manufacturer. It is designed and produced through additive manufacturing that replaces the tibial component and the polyethylene in such a way as to reduce the pieces that are part of the prosthetic assembly to only two: the femoral and the tibial (the so-called “two-component knee prosthesis”). The cytotoxicity as well as the live/dead tests carried out on a series of biomaterials guarantee the best osteointegration of the studied material. The finite element simulation method guarantees the stability of the material before a load of 2000 N is applied in the bending angles 0°, 30°, 60°, 90°, and 120°. Thus, the non-metallic prosthetic material and approach represent a promising alternative for metal-allergic patients.

## 1. Introduction

Total knee replacement surgery is one of the most frequent surgical procedures in orthopedics, with it being the treatment indicated for knee osteoarthritis. Its main objective is to try to improve the quality of life of patients. In the United States, this is the most frequently performed surgical procedure on hospitalized patients [1], and thus 3.48 million total knee arthroplasty (TKA) procedures are expected to be performed annually by 2030 [2]. According to statistics from 2017, this procedure was carried out 130 times per 100,000 inhabitants in Spain. It should be taken into consideration that the cost of the Spanish healthcare system amounted to EUR 1523 per inhabitant in 2018. Moreover, by 2033, life expectancy in Spain shall increase to 83 years for men and 88 for women [3]. These numbers imply that the frequency of this type of surgery shall significantly rise in the coming years. Despite the high rate of success of these interventions, approximately 19% of patients are not contented upon surgery and thus might need to undergo a prosthesis revision surgery [4]. The number of revision surgeries subsequent to primary knee replacement surgery is hence expected to increase. According to [5], in order to reduce the number of revisions of such interventions and to achieve lasting results, it is important to restore joint kinematics, which in turn depends on the implant design as well as on biomechanics [6,7]. The most recent designs are aimed at creating personalized prostheses for the patients. In this regard, additive manufacturing (AM) offers the opportunity to achieve a milestone [8] in the design of very useful custom-made prostheses. Other manufacturing processes, such as compression, injection, or extrusion molding, are processes that are limited by the design as well as the prior manufacture of the molding. Additionally, given the geometry required by the prosthetic elements, it becomes either very expensive or impossible to manufacture. However, AM, as well as 3D printing (3DP) [9], allows the manufacture of any type of complex geometry, whose process implies the addition of layers upon layers of material until the part or the prototype pre-designed in 3D is obtained, using a CAD software tool [10]. According to [11,12], there are several different groups using additive technologies that comply with the aforementioned principle and that are classified by the type of raw material used: liquid phase (for example: Photopolymerization by Stereolithography “SLA” or “SL”), filaments (Extrusion by Fuse Deposition Model “FDM”), laminates (Lamination by Laminated Object Manufacturing “LOM”), or powder bed fusion (Selective Laser Sintering “SLS” or Selective Laser Melting “SLM”, the latter being the most commonly used technology for metallic materials). The process of successively adding material to build a piece involves a high dependency on the machine type and the process parameters. Thus, it is impossible to accurately predict the properties of these materials without making use of specific types of machines and process parameters [11]. Among these additive technologies, the FDM is one of the most widely used methods due to its simplicity, its versatility, and the large number of materials that is able to support/carry [13]. Within the FDM, the properties of the materials are highly influenced by the processing parameters (3D printers) [14]. This is the particular case of the Fortus 450mc FDM printer (supplied by Stratasys) and the ULTEM^TM^ 1010 (U1010) filament material (supplied by Sabic or Stratasys), whose parameters, such as printing temperature, printing speed, scanning path, nozzle diameter, layer thickness, chamber temperature, and filling ratio, guarantee a final piece that meets the technical specifications published by the manufacturer, its only limitation being the yet unknown histological response when implanted in the human or animal body. The U1010 material is a commercial polyetherimide (PEI) with thermoplastic behavior, with the molecular formula: [C37H24O6N2], molecular weight: 592.61 g/g-mol, and density: 1275 kg/m^3^ [15]. Moreover, the U1010 PEI is characterized by an enhanced flow and glass transition temperature (Tg) of 217 °C. It is a biomaterial certified by the Sabic manufacturer, that has been subject to cytotoxicity and live/dead testsHooper et al. [16] used this type of PEI in 3DP in order to manufacture single-use instruments for TKA. In regard to Suffo et al. [17] and Suffo [18], the biomaterial was subjected to tests that were meant to solve the adhesion problems in dental prostheses. Thus, its physicochemical properties deserve an opportunity in the biomechanics of knee prostheses.

The other thermoplastic materials, which can be used for knee prosthesis prototypes in AM, are biocompatible, warranted by the manufacturer, but do not offer suitable mechanical performance to replace current materials based on metallic alloys of Cr–Co–Mo (Vitalium) or titanium [19]. In regard to [16,20], acrylonitrile butadiene styrene (ABS M30i) and polylactic acid (PLA) are two examples of biocompatible materials that are guaranteed by the manufacturer Stratasys, but whose tensile modulus (2300 MPa) is lower than that held by poly aryl ether ketones (PAEKs), poly ether ether ketone (PEEK), or PEI [14], which have an elastic modulus that is similar to human bones [21]. However, there are many types of PEEK available on the market and the lack of knowledge of their real composition adds uncertainty. Keeping the latter mentioned fact in mind, an alternative is to use other materials with the mechanical rigidity that a PEI type guarantees, such as ULTEM^TM^ 9085 or 1010 (biocompatible), which reaches 2800 MPa.

Ultra-high molecular weight polyethylene (UHMWPE) is a synthetic thermoplastic polymer that has been used for over 50 years in total knee arthroplasty due to its good mechanical properties and its well-tolerated behavior, especially friction and wear [22]. Precisely wear, loosening, instability, and infection are the main reasons for TKA revisions according to Sharkey et al. [23], even though Schroer et al. [24] state that 50% of revisions are caused by instability or infection. The wear of PE is directly related to the size of the contact areas and the magnitude of the contact [25]. The wear of PE produces wear particles around the knee implant that can lead to osteolysis, which can consequently lead to the loosening of the implant in the long run [5].

Biocompatibility is a property of a material that measures the ability of a material to resist bacterial colonization in the host, i.e., biocompatible material (biomaterial); it can coexist with human tissue without causing any undesirable or inappropriate effects [26]. To be used as a biomechanical prosthesis, it is necessary for the biomaterial to have at least bioinert behavior, although it is much better if it is bioactive. Bioinert materials are those that do not cause a reaction in the body (commercially pure titanium “cpTi”, PEEK), whereas the hydroxyapatite “HA” ceramics, which are glass ceramics [27] are bioactives that stimulate the tissue or bone growth. Some metals are common materials used in the biomedical field for implants and prosthesis. Their high tensile strength and fatigue characteristics make them suitable for knees and hips [28]. However, metals have limitations related to corrosion, which can lead to toxicity or hypersensitivity reactions that finally produce the rejections referred to above. To consider a biomaterial suitable for implantation, it is not enough for it to be biocompatible; it must comply with cytotoxicity and osteoinduction. For the percentage of the population rejecting metal prostheses, whose causes could be attributed to their special allergy to metals, a new knee prosthesis based on U1010 material is proposed [29].

**Figure 1:**
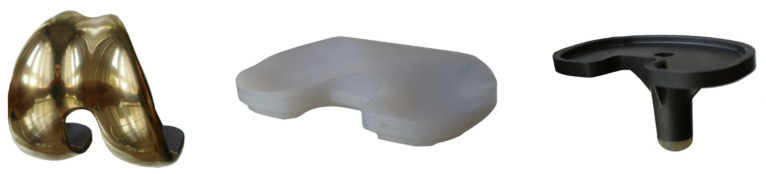
Some parts of cemented knee prothesis, from left to right: femoral component, polyethylene, tibia component [31].

Finally, prior to their manufacture, these prototypes of knee prostheses (the tibial and the femoral parts) must undergo structural tests to confirm a similar performance to the current ones based on metallic materials (titanium or Cr–Co alloys), evaluating the load states caused by the different flexion angles of the knee by means of Finite Element (FE) methods (FEM) [13]. This study focuses on the design of the sliding prosthesis, which is the one used in the vast majority of cases. As can be seen in Figure 1, sliding prostheses are made up of four parts, although there are only three that influence the stability and biomechanical behavior of the set—the fourth part is the patellar button, even though it is not commonly implemented by all surgeons [30].

**Figure 2:**
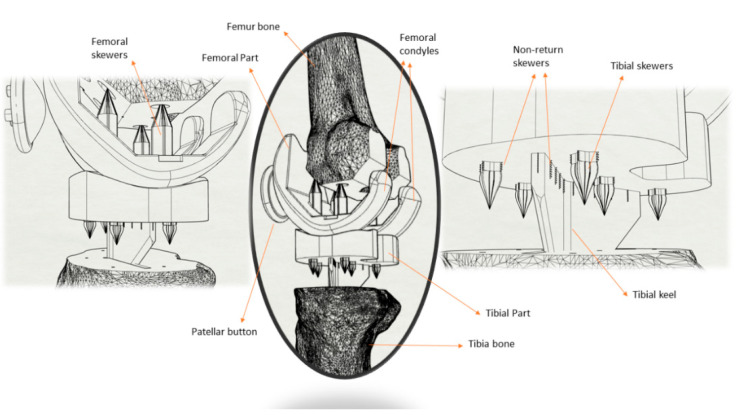
Characteristics of the new bicomponent knee prosthesis proposal.

Starting from the three parts that make up the real prosthesis, an alternative composed only of two parts is proposed (“two-components knee prosthesis”) by fusing the tibial piece with the UHMWPE insert, both with the same material. As shown in Figure 2, the tibial and femoral parts are redesigned using a CAD software tool in order to be better inserted into the bone without the need for cemented material, finally adding some skewers and sawtooth reinforcements.

## 2. Materials and Methods

### 2.1. Description of the Materials

All parts that make up the prototype of the prosthesis are made by Stryker® Orthopaedics, and especially its Triathlon model ® Knee System. Table 1 summarizes the data sheet of the U1010 material produced by the manufacturer Sabic that serves to assess the reliability of the samples of this study. There are three build orientations of coordinate axes of samples shown in the data sheet. The parts were printed under standard parameters and default fill densities. For comparison purposes, other materials were used for the live/dead tests: two made of PEEK: Ensinger (PEEK-E) and Zircozhan (PEEK-Z); Ti–6A1–4V by Biomed, an agrocomposite based in polylactic acid (PLA-CB) with an amount of Carbocal®; and sugar beet subproducts, as used in reference [32].

### 2.2. Test Methods 

Prosthetic parts and 3D bone models were designed using the CAD software tool CATIA V5 (Dassault Systèmes), as shown in Figure 2. In 3D bone models, flat sections are made parallel to those arranged in the prosthetic parts, as stated in the manufacturer’s surgical guide. These sections include holes and grooves to insert the skewers (6 in total) that favor the prosthesis–bone fixation, simulating an embedment (without degrees of freedom). Finally, due to the progressive change in the center of movement, the incorporation of the tibial keel in the shape of a delta wing avoids problems with micromobility when walking.

#### 2.2.1. Parts and Specimens Additive Manufacturing

**Figure 3:**
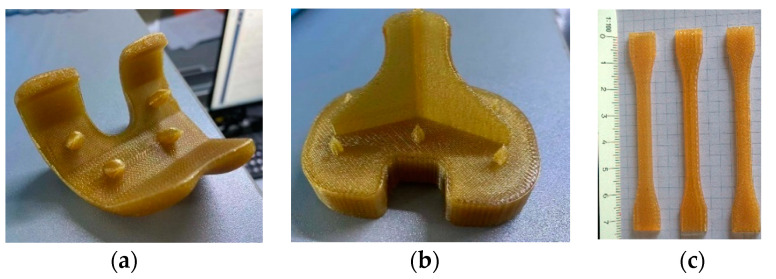
Parts manufactured by FDM technology: (**a**) femoral part; (**b**) tibial part; (**c**) 1BA specimens for tensile tests (XY-XZ-YZ orientation).

The 3D model prosthetic parts and a series of specimens (n = 3) were exported for their manufacture with U1010 biocompatible filament supplied by Sabic and adapted for a Fortus 450MC machine by Stratasys. A T14 type nozzle (0.254 mm) was used for fabrication, whereas the same material was used for the support piece, using a T16 type nozzle (0.254 mm). Figure 3a,b show the final aspect of the parts built using additive manufacturing FDM techniques. The supporting material was removed and the tips of the skewers were sharpened.

#### 2.2.2. Tensile Test

The mechanical properties of the printed materials were analyzed through specimens built specifically for each orientation, obtaining an average. Figure 3c shows test sample 1BA materials obtained following the ISO 527-2:2012 standard for tensile testing. The test was performed at a speed of 5 mm/min with three samples in each orientation.

#### 2.2.3. FEM Simulation 

Three-dimensional parts were modeled by performing an exhaustive measurement on the real references. From the obtained models, the parts were exported to the FEM simulation tool (ANSYS/Academic Research of ANSYS Inc., Houston, PA, USA) for analysis in conformity with the ISO 14243-3: 2009 standard [33], to adapt the loading and displacement conditions according to the references [5,25,34], while considering the U1010 physical properties in replacement of UHMWPE. The physical properties of the U1010 material implemented in the simulations were collated in the tensile test described in the previous section.

**Figure 4:**
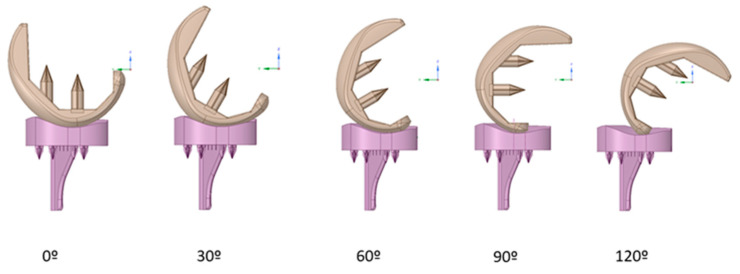
Bending angles considered in the analysis.

This ISO standard suggests that the load distributions for different bending angles: 0°, 30°, 60°, 90°, and 120°, should be taken into account (Figure 4).

**Figure 5:**
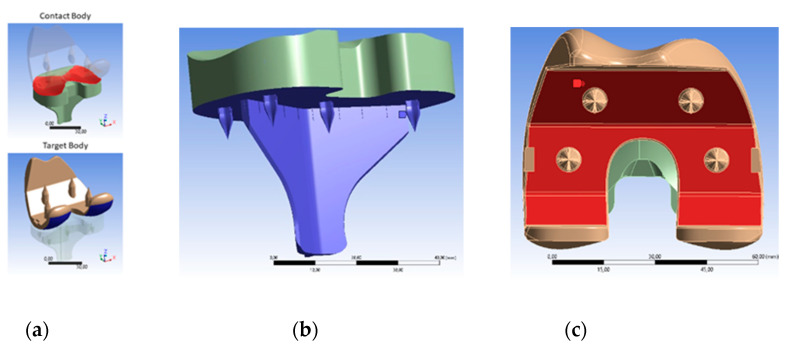
Main characteristics considered in the pre-processor: (**a**) contact properties; (**b**) definition of the fixation; (**c**) definition of the applied force.

Finite element simulations were carried out to analyze the contact interactions between the femoral and tibial components. The appropriate loading and boundary conditions were applied to the femoral and tibial tray components according to the ISO standard [30,35]. In this particular case, a structural static analysis was carried out for each angle considering a rough-type contact between the two parts and applying a friction coefficient of 0.04 [5,32]. It is possible to apply another type of dynamical analysis, but at first, it is intended to prove the stability, reliability, and performance of the U1010 material to external loads applied normally in this new two-component prosthesis. Contact surfaces are shown in Figure 5a. Due to the high characteristics of the rough contact, the pure penalty method is used. This method is recommended for frictional or rough contacts because it can fade out the influence of contact parameters on most of the output parameters [36,37]. A tetrahedron type mesh with an element size of 0.7 mm was applied.

For each movement initiated by a person, when ascending stairs or when squatting, the femur part was rotated about the flexion axis as much as the flexion angle required for the activity. Additionally, it was also rotated about the pivotal axis in order for the femur to be in contact with the tibia part, as expected from a natural knee. Figure 5b shows the fixed support that is assigned on the bottom surface of the tibial part, which is constrained in all directions at the distal end. Figure 4c shows the axial load that is applied to the upper surface of the femoral part, mimicking the bone–implant condition and a body two-fold heavier than 80 kg (2000 N). The linear elastic model is considered for its simplicity and computational efficiency; moreover, the technical specifications of U1010 were followed.

#### 2.2.4. Cell Viability Assay (Ratio Live/Dead) 

The biocompatibility of U1010 was tested with human osteoblastic primary cell cultures (COPH, cells purchased from PROMOCELL as HOB®), since these are the ones that are not genetically modified or resulting from tumors. COPH cultures were grown in osteogenic media and viability/cytotoxicity assays were performed over 7 days. In this assay, called live/dead, dead cells and live cells were labeled with EthD-1 and Calcein AM, respectively. As a negative control, prior to the labeling process, the COPH cultures were incubated with 70% methanol for 30 min. As a positive control, COPH cultures were used without any treatment. First, the culture medium was removed, cells were subsequently washed with PBS, and finally the labeling solution was added. After a 30-min incubation at room temperature, cells were observed under fluorescence microscopy. ImageJ software was used to analyze the intensity of the different fluorescences, and the relationship between both markers was estimated [38,39,40].

#### 2.2.5. Penetration and Fixation Test

**Figure 6:**
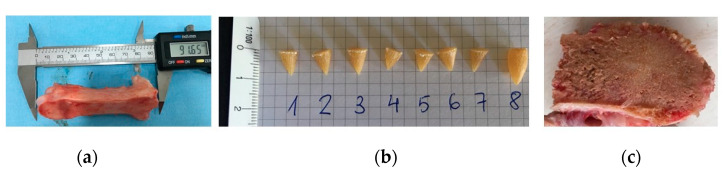
Penetration assay in the bone: (**a**) pork bone used; (**b**) skewer geometry; (**c**) section of the cancellous bone where the skewers will be inserted.

In order to verify that the U1010 material does not present difficulty in penetrating and fixing the trabecular bone, an exclusive test was carried out. Skewers with a similar geometry to those presented in the prosthesis prototype (Figure 6a) were made. The number 8 skewer was slightly larger than the others in order to check a skewer with a similar geometry to those included in the femoral part. By means of the common tools used in TKA, a pork bone that was acquired in a supermarket was sectioned to insert the skewers into its cancellous bone (Figure 6b,c). The trial was carried out by the surgeon co-author of this study according to surgical protocol “minimally invasive surgical” (MIS) by StrykerTM [31]. To confirm the fixation of the skewers without causing internal micro-tears in the bone or the detachment of the U1010 material, cone-beam computed tomographic (CBCT) scans were acquired using CS 9000 3D (Carestream Dental Rochester, NY) [41]. Exposure parameters were estimated at 90 kV and 8 mA, assuming a spherical voxel size, and the field of view was 360° around the pierced bone. The interior distances caused by the skewers were measured to export the DICOM images to 3DSlicer free software [42].

## 3. Results and Discussion

### 3.1. Specimen U1010 Tensile Test

**Figure 7:**
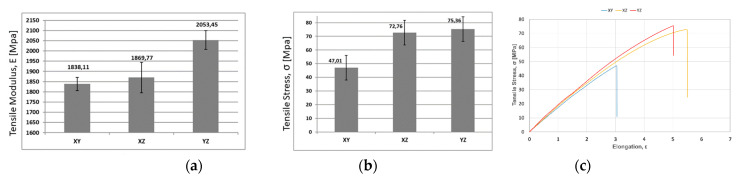
Experimentally measured mechanical properties: (**a**) tensile modulus, E; (**b**) tensile stress, σ; (**c**) stress–strain averaged curves XY, XZ, YZ.

Figure 7 shows the result of the test to compare the mechanical properties published by the manufacturer of the material and those obtained from the experiment. As shown in Figure 7a,b, there is an increase in both tensile modulus “E” and tensile stress “σ” in the XZ (on-edge) and YZ (vertical) orientations.

However, the experimental test demonstrates that the orientation showing the greatest elongation is XZ, reaching up to 5.5%, which contradicts the values published by the manufacturer. Orientation YZ presented 5% compared to XY, which only reached 3%. 

The tensile test confirmed a slight decrease in the mechanical properties (tensile modulus and tensile stress) of U1010 published in the manufacturer’s data sheet (Table 1), as happened in the test carried out in the reference [18]. This shows the good repeatability between the different Fortus-U1010 combinations. In this way, the values that will be used in the FEM simulation (tensile modulus “E” and tensile stress “σ”) will be those obtained here. 

### 3.2. FEM Simulation

The tibio-femoral contact area is the area that accumulates the most stress in the entire prosthetic joint and can reach values that make the material break [43]. In addition, to guarantee its stability, a minimum contact area is necessary [44], hence the importance of simulate their behaviour. To carry out an evaluation of the results, the following characteristic parameters of the simulations will be used according to [45], considering that the present case was applied to U1010 PEI and the cited references used metallic materials and UHMWPE.

Frictional Stress: The frictional stress is the sum of stress components acting along the two XY axes and is a result of the tendency of the two contacting surfaces to slide against each other. In frictional contact, the two contacting surfaces can carry shear stresses up to a certain magnitude across their interface before they start sliding relative to each other. The Coulomb friction model defines an equivalent Shear Stress τ [46,47], at which sliding on the surface begins as a fraction of the contact pressure p (τ = µp + COHE, where µ is the friction coefficient and COHE specifies the cohesion sliding resistance). Once the shear stress is exceeded, the two surfaces will slide relative to each other. This state is known as sliding. The sticking/sliding calculations determine when a point transitions from sticking to sliding or vice versa. Von Mises Stress is used to evaluate the yield zones of the material under contact pressure, and Penetration measures the gap produced by the force exerted on the contact being proportional to it. 

**Figure 8:**
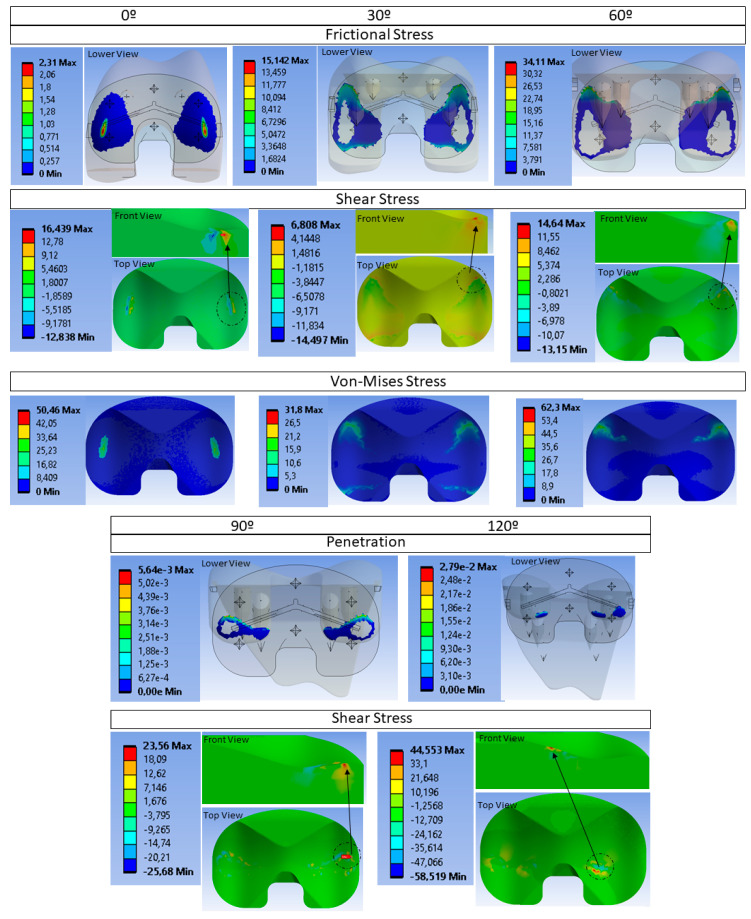
Results of the FEM simulation. (**Top**) Stress frictional (lower femoral part viewpoint of the observer), shear stress and von Mises stress (top tibial part viewpoint of the observer) to flexion angles 0°; 30°;60°. (**Bottom**) Penetration contact and shear stress to 90°;120°.

Figure 8 shows the contact stress and von Mises stress for 0° and 60° of flexion when ascending stairs and at 90°–120° of flexion when squatting in the condylar contact area [6]. 

In the first flexion angles, the contact stress is the highest during the ascent of stairs and during squatting along with the frictional stress and von Mises stress, in agreement with [5]. Under no circumstances is the elastic limit of the material U1010 exceeded, determined in the tensile test (75.36 MPa). Both the frictional stress and penetration are distributed along the posterior–lateral part of the tibial tray, right in the existing barrier to prevent extreme sliding. These results coincide with those obtained by [5,44]. The stress gradient also coincides with that study, evidencing high peaks in the same posterior areas of the tibia. The 0°–60° interval accumulates the largest contact surface (higher than 120 mm^2^). Surprisingly, the U1010 biopolymer has the ability to resist loads twofold higher than previous references without collapsing.

Due to large internal–external rotation during the ascent of stairs, the contact area between tibial and femoral inserts would decrease and hence the stress would increase. A 60° bending angle occurs during the ascent of stairs and constitutes an extreme test that is overcome, as the von Mises stresses in that area are lower than the breaking limit, with the maximum stress values not even occurring in the tibial tray.

Squatting is one of the bodily movements that results in higher stresses on the knee implant due to the higher degrees of flexion, resulting in different contact areas. As the flexion angle increases, the contact decreases, so the peak contact stress and von Mises stresses would increase compared to movements with a lower flexion angle. The contact pressure and frictional stress increase significantly when squatting, as compared to contact stress on the level of walking and the ascent of stairs, due to the higher flexion angle and smaller contact area. 

The maximum penetration value in the contact does not exceed 30µm and occurs at the angle of 120°. Although the stress value triples in the maximum angles, the prosthesis would not suffer deformation since it would be held by the ligaments [6], and these are not relevant results to take into account [5]. The peak shear stress reaches its highest value about 1 mm beneath the articulating surface, as depicted in Figure 6 (between 0° and 60°). This result coincides with previously referenced studies and a decrease in the pressure-stress in the 60° to 90° contact is not observed, unlike what is evidenced by [48].

### 3.3. Live/Dead Test

**Figure 9:**
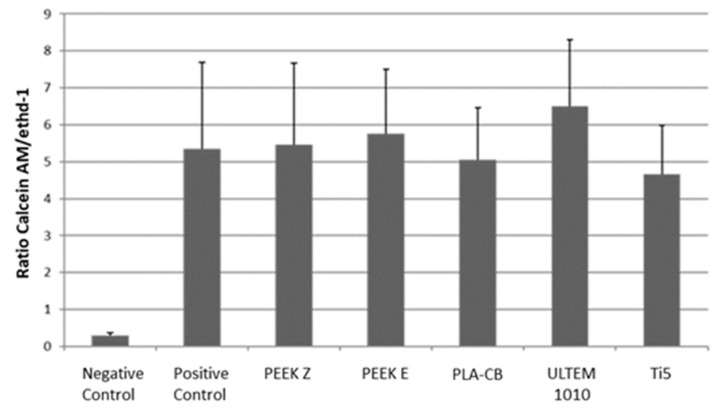
Ratio of live/dead cells of the biomaterials after 7 days.

According to the results obtained and presented in Figure 9, none of the tested materials interfere with the growth and cell viability of human osteoblasts, making them optimal for future bioactivity tests in cultures with human osteoblasts. This conclusion is supported by the comparison between the positive control and the different cultures for each material. 

The negative control is COPH treated with a cytotoxic agent known as 70% methanol for 30 min, while similar results are obtained between the positive control and the COPH treated with the different materials. In particular, the U1010 material has an interesting positive effect on the COPH cell viability when this treatment is compared with the positive controls, exhibiting a ratio above 6. The other materials (PEEK Z, PEEK E, PLA-CB, and Ti) have a similar cellular compatibility to COPH in a normal growth state (positive control). In the absence of continuity in follow-up, at 7 days, it is possible to exclude the cytotoxic effects of these materials on human osteoblast cells, as observed in the negative control (70% methanol).

### 3.4. Penetration and Fixation Test

The hardness of the trabecular and cortical bone is well known [49]. However, in non-isotropic parts manufactured using FDM, this property is difficult to determine empirically. In the absence of standardization to solve this problem, it was decided to reproduce the hammer blow that the traumatologist performs when they try to fix the prosthetic parts to the bones. If the U1010 skewers are well fixed to the bone and neither element breaks (skewer nor bone), the material will be valid for use in prostheses.

**Figure 10:**
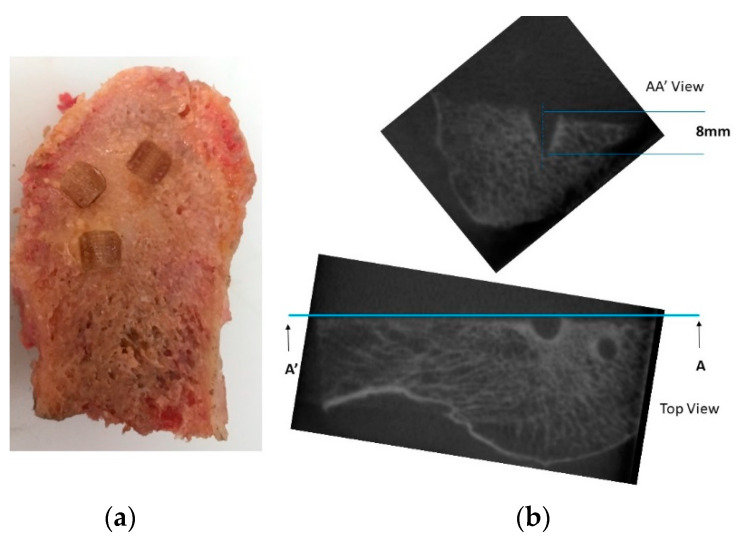
Penetration assay images and fixation of skewers to the bone: (**a**) skewers driven into cancellous bone; (**b**) orthogonal views of the exposure to RX. Top view) Holes formed by the skewers are represented and one of them is sectioned by AA’ cutting plane. AA’ View) Sectional representation that indicates the height of the skewer.

All the skewers that were tested on the bone surface were penetrated to the bottom and firmly fixed by the surgical mallet. The areas where the bone was not hard enough were discarded. As shown in Figure 10a, they were tested in two similar bones with satisfactory results in both cases.

In Figure 10b, images obtained from the bone’s exposure to RX that were taken as a sample are represented. Orthogonal views of the spikes reveal that there were no internal micro-tears in the bone, which remained intact. In addition, there were no detachments of U1010 material.

## 4. Conclusions

This work gives rise to an innovative biomechanical design of two-component knee prostheses manufactured using FDM in a PEI thermoplastic material. Additionally, it shows that because of its structural stability, mechanical properties, and chemical resistance, the U1010 biopolymer works similarly to others, such as titanium or Cr–Co alloys and PEEK, which have been successfully applied in biomedical disciplines for decades. The tensile test on U1010 specimens has empirically revealed that the mechanical properties of the printed material in FDM are inferior to those published by the manufacturer in its data sheet. This fact has coincided with other similar tests using another machine–material combination (Fortus 450-U1010), which reveals good repeatability and confidence. In the absence of other mechanical tests in a bioreactor (stress shielding) [49], the FEM simulations suggest stable behavior against the pressures exerted in contact during flexion joint movements. In none of the simulated load hypotheses is the elastic limit of the PEI material exceeded, considering the two most severe physical activities: ascending stairs and squatting. Although there is a lack of scientific rigor, the penetration test was enough to simulate surgery in an operating room. The penetration test shows that the U1010 material is perfectly embedded in the bone, which guarantees the fixation of the two parts, femoral and tibial, when joining their bones, femur and tibia, respectively. Despite its limitations, the live/dead test shows that the material does not exert a negative cellular effect after 7 days.

Prior to its definitive implantation in humans, new phases of study are required, particularly those related to (in vivo) clinical trials in animals using the material in order to ensure a long-term performance as good as the one reported in this study. Future work will also focus on the characterization of other types of biomaterials and bio-agrocomposites, such as PLA CB, to assess their potential application in this type of knee and hip prosthesis.

## 5. Patents

Upon submission of the paper, an invention patent of the new U1010 material for knee prostheses has been registered (Spain: P202130869, 17 September 2021).

## Figures and Tables

**Table 1:** ULTEM^TM^AM1010F biomaterial used in this study [15].

Orientations ^1^	XY (Flat)	XZ (on-Edge)	YZ (Vertical)
Tensile Modulus ^2^ (MPa)	2750	2865	2840
Tensile Stress ^2^ (MPa)	34	73	80
Tensile Strain ^2^ (%)	4	3.8	1.3

^1^ the orientation of the coordinate axis (according to [15]—Figure 6). ^2^ v = 5 mm/min.

## Data Availability

This study did not report any data.
